# Biocompatibility and Transplantation Efficacy of the C-Clear Artificial Cornea in a Rabbit Chemical Burn Model

**DOI:** 10.3390/bioengineering10101235

**Published:** 2023-10-21

**Authors:** Ho-Seok Chung, Sanghyu Nam, Ko-Eun Lee, Do-Sun Jeong, Seheon Oh, Jeong-Hye Sunwoo, Hun Lee, Jae-Yong Kim, Hungwon Tchah

**Affiliations:** 1 Department of Ophthalmology, Asan Medical Center, University of Ulsan College of Medicine, Seoul 05505, Republic of Korea; chunghoseok@gmail.com (H.-S.C.); thehyu@gmail.com (S.N.); skymeun@naver.com (K.-E.L.); 72004218osh@gmail.com (S.O.); swjheay@naver.com (J.-H.S.); yhun777@gmail.com (H.L.); jykim2311@amc.seoul.kr (J.-Y.K.); 2 TE BioS Co., Ltd., Cheongju 28160, Republic of Korea; ceo@tebios.com; 3 Center for Cell Therapy, Asan Institute for Life Science, Seoul 05505, Republic of Korea; 4 Department of Ophthalmology, Myung-Gok Eye Research Institute, Kim’s Eye Hospital, Konyang University College of Medicine, Seoul 07301, Republic of Korea

**Keywords:** C-Clear, artificial cornea, rabbit chemical corneal burn model, retroprosthetic membrane

## Abstract

We investigated the bioavailability and stability of a C-Clear artificial cornea in a rabbit chemical burn model. Thirty-six rabbits were divided into a control group (*n* = 16) and a chemical burn group that used NaOH solution (*n* = 20). After lamellar dissection, the central posterior lamella was excised using a 3 mm diameter trephine, and an artificial cornea was transplanted into the lamellar pocket. After 2 weeks, the central anterior lamella was excised using a 3 mm diameter trephine to secure a clean visual axis. We examined the anterior segment of the eyes weekly for 12 weeks after transplantation. Successful subjects whose artificial corneas were maintained stably for 12 weeks were euthanized and underwent histologic examinations. Artificial corneas remained stable for up to 12 weeks in 62.5 and 50% of rabbits in the control and chemical burn groups, respectively. Two rabbits in the chemical burn group showed the formation of a retroprosthetic membrane, and one rabbit with visual axis blockage underwent membrane removal using a Nd:YAG laser. In histologic examinations, adhesion between artificial cornea and peripheral corneal stoma was observed. In conclusion, we confirmed structural stability and biocompatibility of the C-Clear artificial cornea for up to 12 weeks after implantation in control and chemical burn groups.

## 1. Introduction

Corneal injuries, such as trauma and chemical burns, and diseases, such as trachoma and infectious keratitis, are the fifth leading cause of blindness worldwide, with an estimated 4.5 million reported cases annually [1,2]. Currently, the only treatment for permanent corneal opacity is allogeneic corneal transplantation, in which a donor cornea is transplanted into the recipient’s eye. However, because of a shortage of donor corneas, approximately 13 million patients are waiting for allogeneic corneal transplants globally [3]. 

Patients with corneal damage from Stevens–Johnson syndrome, herpes simplex keratitis, chemical burns, concomitant glaucoma, or corneal neovascularization are at high risk for corneal transplantation failure [4,5]. Moreover, repeat corneal transplantation in high-risk patients is associated with shorter graft survival duration and lower transplantation success rates [6,7,8]. Therefore, artificial corneal transplantation could be considered an alternative to allogeneic corneal transplantation in patients with a high risk of corneal transplantation failure. 

The Boston KPro® (Massachusetts Eye and Ear Infirmary, Boston, MA, USA) and AlphaCor® (Argus Biomedical Pty Ltd., Perth, Australia) are the only artificial corneas that have been approved by the FDA for full-thickness transplantations [9]. The Boston KPro® is fabricated using polymethylmethacrylate (PMMA) and was approved for use in 1992; however, its drawback is that it cannot address the issue of the shortage of donor corneas because it requires inserting a partial donor cornea between the front and back plates [10]. The AlphaCor® is fabricated using poly-2-hydroxyethyl methacrylate (PHEMA) and was approved in 2003; production was discontinued because of biocompatibility issues after surgery. Hence, the demand for an artificial cornea composed of a novel material has emerged. Although engineered corneal equivalents using immortalized human corneal cells or cyclodextrins have been introduced, they is insufficient in practical transplantation [11,12].

**Figure 1:**
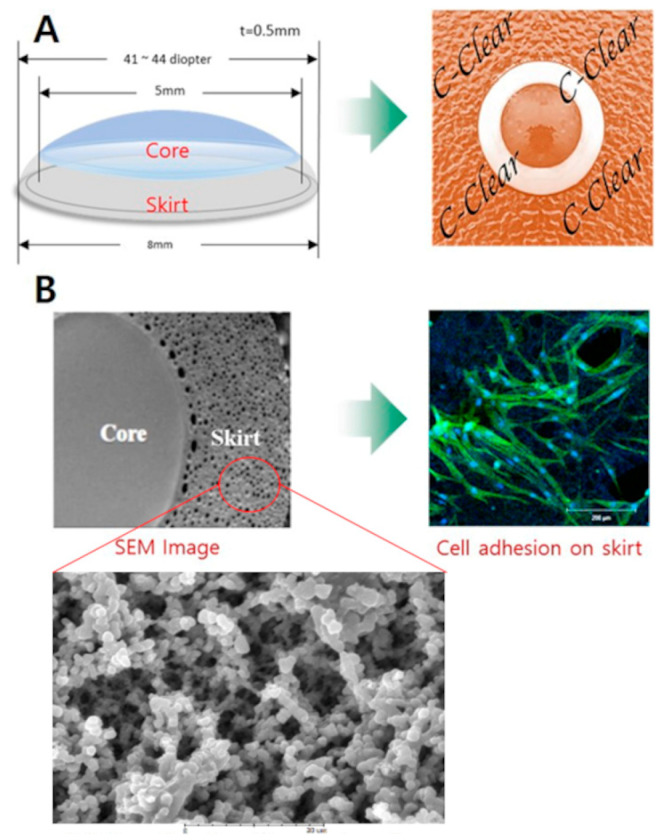
Structure of the C-Clear artificial cornea. (**A**) A schematic diagram provided by the manufacturer depicting the integrated structure and a radius of curvature similar to that of a human cornea. (**B**) The porous nature of the peripheral skirt facilitates the engraftment of the artificial cornea by inducing the migration and proliferation of surrounding corneal cells, which is confirmed via phalloidin and DAPI staining. SEM, scanning electron microscope.

The C-Clear (TE BioS Co., Ltd., Cheongju, Republic of Korea) is a new artificial cornea with a transparent core (diameter, approximately 5 mm) and a peripheral skirt (width, 1.5 mm). The central optical component prevents the adherence of proteins or cells and can, therefore, provide transparency similar to that of the living cornea (Figure 1A). Moreover, the porous structure of the peripheral skirt increases the surface area, allowing for a more secure attachment to the recipient cornea and a reduced risk of displacement of the artificial cornea (Figure 1B). The C-Clear is expected to be safe, biocompatible, and effective in restoring the function and shape of damaged corneas without the need for donor corneal tissue. In this study, we investigated the biocompatibility and stability of C-Clear artificial corneas in a rabbit chemical corneal burn model. 

## 2. Materials and Methods

### 2.1. Animals

Thirty-six white New Zealand rabbits, each weighing between 2.5 and 3.0 kg, were used in this study. The animals were housed in standard rabbit cages under controlled environmental conditions. All experimental procedures conformed to the guidelines of the Association for Research in Vision and Ophthalmology Statement for the Use of Animals in Ophthalmic and Vision Research. This study was conducted in strict accordance with the relevant national and international guidelines regarding animal handling as mandated by the Institutional Animal Care and Use Committee of the University of Ulsan College of Medicine (Seoul, Republic of Korea), which reviewed and approved the animal study protocol (2021-12-272). 

All interventions were performed under anesthesia, and all efforts were made to minimize animal suffering. When required, the rabbits were euthanized using an intravenous injection of potassium chloride solution, and death was confirmed. 

All rabbits were anesthetized using intramuscular injection of a mixture of tiletamine, zolazepam (Zoletil^®^ 50; Virbac Corp., Carros Cedex, France), and xylazine (Rompun; Bayer, Leverkusen, Germany). Thereafter, topical anesthesia was administered using 0.5% proparacaine hydrochloride (Alcaine^®^; Alcon Laboratories, Fort Worth, TX, USA). The rabbits were divided into a control group (*n* = 16) and a chemical burn group (*n* = 20). In the control group, an artificial cornea was transplanted onto the normal rabbit cornea; in the chemical burn group, a chemical burn was made in one eye, and the artificial cornea was then transplanted onto the affected cornea.

### 2.2. Establishment of the Rabbit Corneal Chemical Burn Model

A chemical burn was made on one eye of each subject in the chemical burn group using NaOH solution as previously described [13,14,15]. Briefly, a piece of circular filter paper suitable for the size of the cornea was dipped in 0.5 N NaOH solution and then placed on the center of the cornea for approximately 30 s. After washing with a balanced salt solution (Alcon, USA) and levofloxacin (Cravit®, Santen, Osaka, Japan), 0.1% fluorometholone (Flumetholon®, Santen, Japan) and a polymyxin B sulfate-neomycin sulfate-dexamethasone ointment (Forus®, Samil Pharm, Seoul, Republic of Korea) were administered to the eye. The expected effects of the model were confirmed by observing corneal opacity and neovascularization 3–4 weeks after treatment. We assessed the appropriateness of the chemical burn model using the corneal opacity scoring system previously described [16]. Briefly, 0 = no opacity, completely clear cornea; 1 = slightly hazy, iris and lens visible; 2 = moderately opaque, iris and lens still detectable; 3 = severely opaque, iris and lens hardly visible; and 4 = completely opaque, with no view of iris and lens. After confirming that the corneal opacity score was ≥2, we performed the artificial corneal transplantation.

### 2.3. Transplantation of C-Clear artificial Corneas

**Figure 2:**
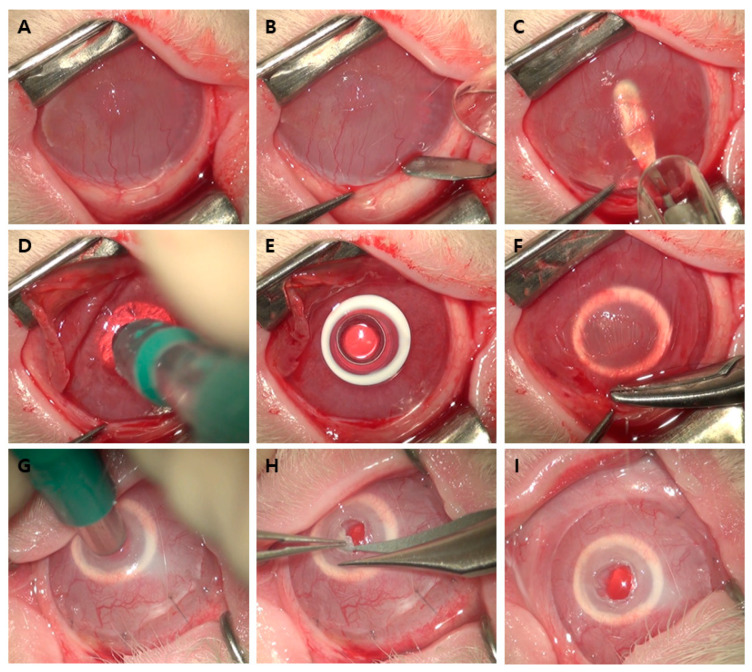
Summary of the artificial corneal transplantation. (**A**) Corneal opacity and neovascularization were observed in the chemical burn group. (**B**) A semicircular incision was made at the cornea 1 mm anterior from the limbus. (**C**) Lamellar dissection was performed using a crescent blade. (**D**) The anterior lamella was folded up, and part of the central posterior lamellar disc was removed using a 3 mm trephine and fine scissors. (**E**) The C-Clear artificial cornea was inserted into the space below the anterior lamella. (**F**) The semicircular corneal incision was sutured using 10-0 nylon. (**G**) Two weeks after transplantation, 3 mm of the central anterior lamella was punched using the same trephine. (**H**) The anterior lamellar disc was removed using fine scissors. (**I**) A transparent central visual axis was observed at the end of the surgery.

Artificial corneal transplantation was performed as follows (Figure 2). A diamond knife was used to make a semicircular corneal incision 1 mm anterior from the limbus (Figure 2B). Using a crescent blade, lamellar dissection was performed at a depth of 250 µm from the corneal incision to secure a space to insert the artificial cornea (Figure 2C). After sufficient dissection, including the central cornea, the anterior lamella was folded up, and part of the central posterior lamella was excised in a circular shape using a 3 mm diameter trephine (Barron Radial Vacuum Trephine®; Katena Products Inc., Denville, NJ, USA) and fine scissors (Figure 2D). The C-Clear artificial cornea was then inserted, with its center aligned as closely as possible with the excised posterior lamina (Figure 2E). The artificial cornea was covered with an anterior lamella while preventing the center of the artificial cornea from being displaced, and the semicircular incision was sutured 4–6 times using 10-0 nylon (Figure 2F). Transplantation was followed by subconjunctival injection of gentamicin (Gentamicin injection®, Shinpoong Pharm, Seoul, Republic of Korea) and dexamethasone (Jeil Dexamethasone injection®, Jeil Pharm, Seoul, Republic of Korea). Two weeks after artificial corneal transplantation, anesthesia was administered in the same manner, and the center of the anterior lamina covering the optic region of the transplanted artificial cornea was removed using the same 3 mm diameter trephine and fine scissors to secure a clean visual axis (Figure 2G,H). 

### 2.4. Anterior Segment and Histological Examinations

After artificial corneal transplantation, the anterior segment of the eye was examined under a light microscope weekly, and corneal opacity, the presence or absence of discharge, and protrusion of the artificial cornea were assessed. All subjects were observed for up to 12 weeks, and subconjunctival injections of gentamicin and dexamethasone were administered at each examination. If keratitis, endophthalmitis, or protrusion of the artificial cornea occurred during observation, the transplantation was considered a failure. Conversely, transplantation was considered successful if the structural stability and optical transparency of the artificial cornea were maintained for up to 12 weeks after transplantation. 

In case of successful transplantation, the animal was euthanized at the 12-week follow-up time point. After the in vivo anterior segment optical coherence tomography (AS-OCT, Casia 2®, Tomey, Nagoya, Japan) examination, the eyeball with the artificial cornea was enucleated. The enucleated eyes were fixed in neutral buffered formalin (3.7% formaldehyde) for 24 h and then embedded into paraffin blocks. The processed tissue was sectioned into 4 μm thick sections, which were then mounted on slides. After deparaffinization, the slides were heated in 0.01 M sodium citrate buffer solution (pH 6.0) at 90–100 °C for 30 min for antigen retrieval. Staining was conducted using a VECTASTAIN® Elite® ABC-HRP Kit (PK-6100, Vector Laboratories, Newark, CA, USA). Briefly, the sections were incubated in BLOXALL® Blocking Solution (SP-6000; Vector Laboratories) for 10 min at room temperature (RT) to block endogenous peroxidase activity and then blocked for 30 min using the normal horse serum provided in the kit. After washing three times for 10 min each, the sections were incubated with anti-alpha smooth muscle actin (α-SMA) primary antibody (1:100; ab18147; Abcam, Inc., Cambridge, UK) overnight at 4 °C. Thereafter, the sections were incubated with the secondary biotinylated horse anti-mouse IgG antibody provided in the kit at RT for 30 min. Staining was visualized using a DAB substrate kit (SK-4100; Vector Laboratories, CA, USA). Sections were also stained with hematoxylin and eosin (H&E). All sections were then examined using a scanning electron microscope (S-4500; Hitachi, Inc., Tokyo, Japan).

## 3. Results

### 3.1. Maintenance of Artificial Cornea up to 12 Weeks

The transplantation procedure was completed without complication for all 36 rabbits. In the control group, the artificial cornea was successfully transplanted and maintained until 12 weeks after transplantation in 10 of the 16 rabbits (62.5%). Protrusion of the artificial cornea, defined as transplantation failure, occurred in the remaining six subjects (observed at an average of 7.2 weeks {range, 3–12 weeks} after the procedure).

In the chemical burn group, the artificial cornea was successfully transplanted and maintained until 12 weeks after transplantation in 10 of the 20 rabbits (50.0%). The other 10 were classified as cases of transplantation failure—three eyes showed signs of infection (observed at an average of 5.3 weeks {range, 1–8 weeks} after the procedure), and seven eyes had protrusion of the artificial cornea (observed at an average of 4.3 weeks {range, 2–7 weeks} after the procedure), with three of these seven eyes also experiencing corneal melting. 

**Figure 3:**
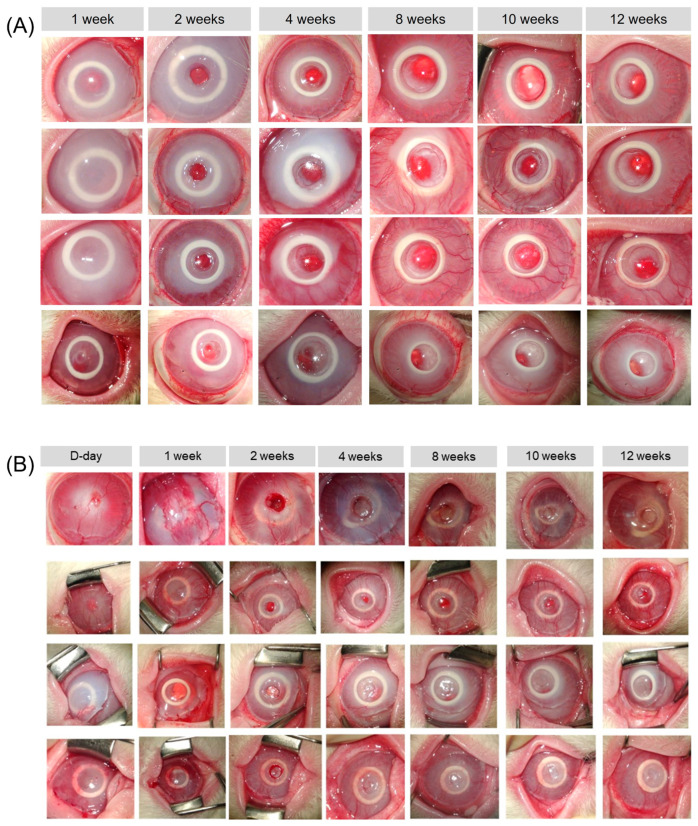
Representative anterior segment micrographs of four rabbits from each group, (**A**) control group and (**B**) chemical burn group rabbits, with stable artificial corneas in the follow-up period (up to 12 weeks).

**Figure 4:**
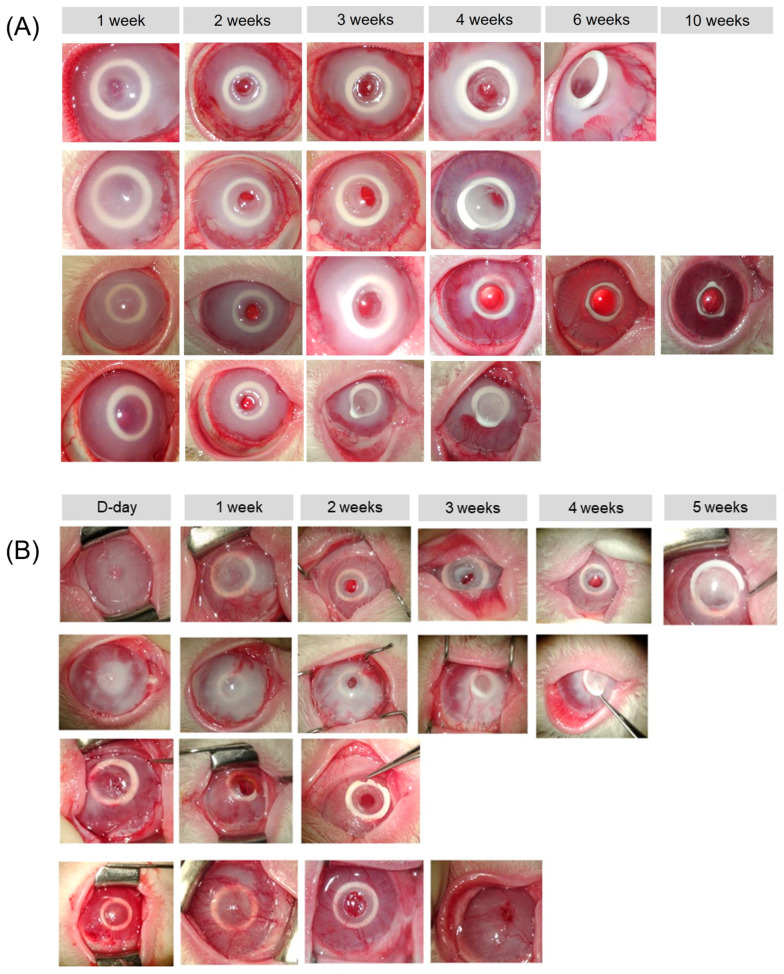
Representative anterior segment micrographs of four rabbits from each group, (**A**) control group and (**B**) chemical burn group rabbits, during follow-up that had failed transplantation due to protruding artificial corneas. The implanted artificial corneas protruded at an average of 7.2 weeks in the control group and 4.3 weeks in the chemical burn group.

In two eyes of the chemical burn group, a retroprosthetic membrane was observed at 3 and 7 weeks after transplantation, respectively. In one case, the membrane was removed using a Nd:YAG laser because the visual axis was blocked. Figure 3 shows anterior segment micrographs taken from rabbits in both groups with artificial corneas maintained until 12 weeks. Figure 4 shows anterior segment micrographs taken from rabbits with transplantation failure in both groups.

### 3.2. AS-OCT Findings

**Figure 5:**
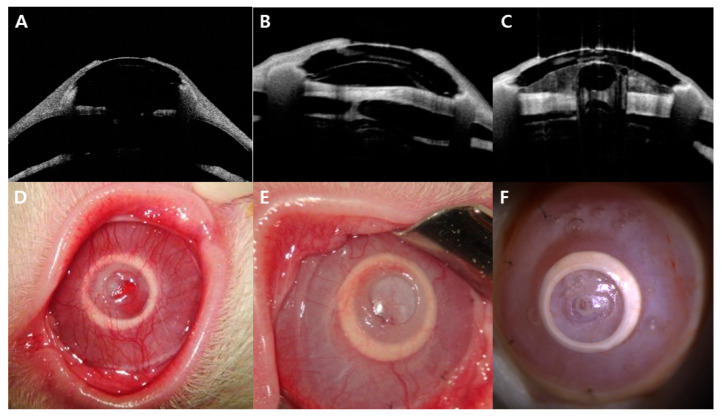
Anterior segment optical coherence tomography images and anterior segment images corresponding to the optical coherence tomography images after C-Clear artificial corneal transplantation. (**A**,**D**) Representative image at 12 weeks; the artificial cornea is successfully attached to the surrounding corneal tissue, with a well-maintained anterior chamber. (**B**,**E**) Representative image of a cornea with a retroprosthetic membrane at 3 weeks after transplantation. A thick membrane is observed behind the transparent artificial cornea. (**C**,**F**) After membrane removal using Nd:YAG laser, the central portion of the thick membrane was successfully removed.

Figure 5 shows AS-OCT images from successful transplantation cases in both groups. The artificial cornea is stably positioned in the groove created in the recipient corneal stroma, and the central part of the cornea is composed solely of the artificial cornea, with no recipient corneal tissue present (Figure 5A). A thick retroprosthetic membrane behind the artificial cornea can also be observed in one representative AS-OCT image (Figure 5B). The central part of this membrane was removed using a YAG laser (Figure 5C). Throughout the observation period, the central optic area of the artificial cornea remained transparent and free of haziness or opacity, except in cases where a retroprosthetic membrane was present.

### 3.3. H&E Staining and α-SMA Staining Results

**Figure 6:**
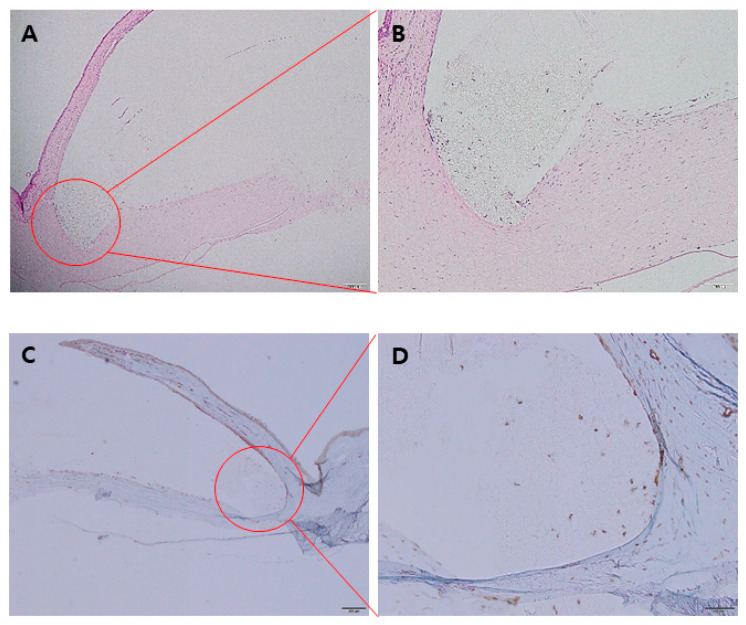
Representative histological images obtained from one rabbit in the chemical burn group, euthanized after 12 weeks of follow-up. (**A,B**) Hematoxylin and eosin staining confirmed that the artificial corneal skirt is successfully attached to the corneal stroma, and corneal cell migration to the porous artificial corneal skirt had occurred ((**A**): magnification 40×, (**B**): magnification 100×). (**C**,**D**) Alpha smooth muscle actin staining in the same subject confirmed that the cells migrating from the corneal tissue are of fibroblast origin ((**C**): magnification 40×, (**D**): magnification 100×).

Histological examination confirmed the stable positioning of the artificial cornea within the created lamellar pockets. Furthermore, cell migration from surrounding corneal tissue to the porous skirt was observed in representative histological images, indicating firm attachment of the artificial cornea to the recipient cornea (Figure 6A,B). Additionally, cells that had migrated from the recipient corneal tissue to the porous skirt were positively stained for α-SMA (Figure 6C,D).

## 4. Discussion

Our study demonstrated that the C-Clear artificial cornea remained transparent and stable for up to 12 weeks in 62.5% of rabbits with a normal cornea and 50.0% of chemical burn model rabbits. Furthermore, we observed cellular proliferation from the surrounding corneal tissue to the peripheral skirt of the artificial cornea, indicating attachment to the surrounding tissues in both groups. This study was meaningful in that a newly developed artificial cornea can restore visual acuity in patients with refractory corneal disease without the need for donor corneal tissue. We plan to conduct a primate eye study and clinical trial, pending permission from the Koran Ministry of Food and Drug Safety, based on the outcomes of this research.

Although allogeneic corneal transplantation is a good treatment option that can be used to treat refractory corneal disease, it does have certain disadvantages that are not easily resolved, such as the shortage of donor corneas and allogeneic rejection. Moreover, high-risk patients with limbal stem cell deficiency, chemical burns, herpes keratitis, and Stevens–Johnson syndrome have a high possibility of transplant failure [4,5,6,7]. Among these conditions, chemical burns of the cornea have been treated with amniotic membrane and limbal stem cell transplantations along with penetrating keratoplasty. However, artificial corneal transplantation is considered feasible when corneal clarity is not restored [17,18,19].

The Boston KPro artificial cornea, which has been most widely used in refractory cases so far, cannot solve the problem of donor cornea shortage [9,10]. The AlphaCor is a soft-type corneal prosthesis inserted between remaining corneal tissues and can improve visual acuity without the need for donor corneal tissue. However, maintenance rates after AlphaCor implantation were reported to be 92, 80, and 62% at 6 months, 1 year, and 2 years, respectively [20]. Stromal melting of recipient cornea and optical surface deposits were also major adverse effects [9]. Stromal melting occurred in 27–58% of cases after the artificial corneal transplantation, and device protrusion occurred in 65% cases [20,21]. Therefore, in the present study, we investigated the bioavailability and stability of the new C-Clear artificial cornea designed to overcome the disadvantages of the AlphaCor keratoprosthesis in both normal and chemical burn model rabbit corneas. 

This newly developed C-Clear artificial cornea is a soft-type corneal prosthesis that does not cause aqueous humor blockage, which occurred with the Boston KPro, thus reducing the possibility of sterile keratolysis due to decreased nutritional support. In contrast to Alphacor, composed only of PHEMA, the C-Clear prosthesis is fabricated by polymerizing both PMMA and PHEMA and has a thinner structure. The polymerization ratio of the two components of C-Clear is PHEMA 91.0%, PMMA 9.0% in the central optic region, PHEMA 82.2%, and PMMA 17.8% in the peripheral skirt. In our experiment, the peripheral skirt of C-Clear demonstrated a 3.2 times higher ultimate tensile strength than that of AlphaCor. Furthermore, it maintained an elongation force of up to 200%, showcasing excellent material stability. Scanning electron microscopy images show that the AlphaCor has small size pores (diameter, 1–10 μm) in the skirt region [22]. The C-Clear artificial cornea has more pores of diameter 30–70 μm in the peripheral skirt, and both the number and size of the pores are increased compared to that of AlphaCor (Figure 1B). This promotes cell migration and proliferation, thereby increasing the adhesion of the artificial cornea to the surrounding tissues, resulting in an increased retention rate.

In 62.5% of rabbits with normal corneas, the artificial cornea was stably maintained for 12 weeks; in 37.5%, artificial corneal protrusion occurred at an average of 6.4 weeks. In 50.0% of the chemical burn model rabbits, the artificial corneas remained stable for 12 weeks; thus, a slightly lower success rate and earlier protrusion than in rabbits with normal corneas were observed. Considering that artificial corneal protrusion occurs because the anterior lamellar flap is too thin to withstand the posterior pressure or because fibroblast proliferation is insufficient, proper formation of a lamellar flap with a thickness of 250 μm or more is important to avoid protrusion. In the chemical burn model rabbits, the tip of the crescent blade was poorly visible, and a thin anterior lamella may have formed because of corneal opacity and neovascularization, which may have led to a high protrusion rate. However, the newly developed artificial cornea is designed to fit the human cornea, which has a thickness of approximately 500 μm. Protrusion is likely to have occurred in white New Zealand rabbit corneas (thickness of approximately 350 μm) because of the structural differences between humans and rabbits [23]. Flap formation with a constant thickness using a femtosecond laser can be considered; furthermore, additional information on structural stability or protrusion rates for the primate or human eye will need to be obtained, which is currently in progress. 

On histologic examination, we observed cell migration from the corneal stroma to the artificial cornea; moreover, H&E staining showed that the skirt of the artificial cornea had adhered to the surrounding corneal stroma. In a previous study by our group, we observed an increase in cell migration over time when comparing rabbits sacrificed at 4, 8, and 12 weeks, respectively [24]. Using α-SMA staining to determine the origin of the cells migrating from the corneal tissue, we confirmed that the migrating cells were fibroblasts. A previous study reported that the multi-porous skirt prevents the protrusion of the artificial cornea by improving biological adhesion with corneal tissues [22]. The new artificial cornea also increases the size and number of pores to further facilitate the firm adherence of the skirt to the surrounding tissues without inflammation in vivo. Since no serious structural abnormality was observed while the C-Clear artificial cornea was maintained stably for 12 weeks, encouraging results in primate and human eyes can be expected.

Retroprosthetic membrane formation and optical precipitates were serious complications noted with the AlphaCor [9]. In this study, two eyes (10%) in the chemical burn group showed the formation of a retroprosthetic membrane. One eye underwent membrane removal using Nd:YAG laser; the other eye was observed without treatment, sparing the optical center. Retroprosthetic membrane formation was observed in 32–65% of patients after Boston KPro type 1 implantation and 13.0% of patients after AlphaCor implantation in previous studies. In our study, it occurred with a frequency similar to AlphaCor [20,25,26]. Histologically, the retroprosthetic membrane comprises collagen fibers and fibroblast-like cells, with no epithelial or inflammatory cells. It is believed to be formed due to attempts to heal from the cut edge of the corneal posterior lamella, which is similar to the wound healing process [27]. Since the degree of wound healing and retroprosthetic membrane occurrence are expected to differ for each species, future studies on retroprosthetic membrane formation incidence and risk factors in primates and humans should be conducted.

No precipitate on the central optic of the artificial cornea was observed in any case in this study during the 12-week follow-up period. In previous reports, most cases of calcific precipitates occurred after 12 weeks of implantation and were related to topical medications or systemic diseases [28]. However, this study may not be sufficient to make conclusions regarding these issues due to the relatively short observation period of 12 weeks and the differing structure of rabbit corneas compared to those of humans. Therefore, long-term observations in primate and human eyes are needed to address these aspects.

This study had some limitations. First, we did not evaluate intraocular pressure after implantation of the artificial cornea. Second, since AS-OCT images were not taken serially, semi-quantitative data regarding structural stability, such as the timepoint of residual stromal proliferation to the skirt portion, could not be obtained. Finally, we did not perform ocular surface staining of the chemical burn model eyes before transplantation. There were some cases where central epithelial defects occurred after the chemical burn model was created. However, we did not evaluate the central epithelial defect area because the central 3 mm of the anterior lamella would be excised and removed 2 weeks later.

In conclusion, we confirmed the biocompatibility and structural stability of the C-Clear artificial cornea for up to 12 weeks after implantation in rabbits, both in the control and chemical burn groups. Based on these results, we are conducting artificial cornea implantation experiments in primates and humans. If clinical studies confirm the promising results, it could become a much-needed solution for patients with refractory corneal disease waiting for allogeneic corneal transplantation. Additionally, it could benefit patients in high-risk groups who have a low transplantation success rate.

## Data Availability

The datasets generated during and/or analyzed during the current study are available from the corresponding author on reasonable request.
